# A Multimetric Benthic Macroinvertebrate Index for the Assessment of Stream Biotic Integrity in Korea

**DOI:** 10.3390/ijerph9103599

**Published:** 2012-10-15

**Authors:** Yung-Chul Jun, Doo-Hee Won, Soo-Hyung Lee, Dong-Soo Kong, Soon-Jin Hwang

**Affiliations:** 1 Department of Environmental Science, Konkuk University, Seoul 143-701, Korea; Email: eco072@empal.com; 2 Doohee Institute of Ecological Research, Korea Ecosystem Service Inc., Seoul 153-768, Korea; Email: drdoogy@kes.re.kr; 3 The National Institute of Environmental Research, Inchon 404-170, Korea; Email: ishnier@korea.kr; 4 Department of Biology, Kyonggi University, Suwon 443-760, Korea; Email: dskong@kyonggi.ac.kr

**Keywords:** multimetric index, benthic macroinvertebrates, biological integrity, stream health, bio-assessment

## Abstract

At a time when anthropogenic activities are increasingly disturbing the overall ecological integrity of freshwater ecosystems, monitoring of biological communities is central to assessing the health and function of streams. This study aimed to use a large nation-wide database to develop a multimetric index (the Korean Benthic macroinvertebrate Index of Biological Integrity—KB-IBI) applicable to the biological assessment of Korean streams. Reference and impaired conditions were determined based on watershed, chemical and physical criteria. Eight of an initial 34 candidate metrics were selected using a stepwise procedure that evaluated metric variability, redundancy, sensitivity and responsiveness to environmental gradients. The selected metrics were number of taxa, percent Ephemeroptera-Plecoptera-Trichoptera (EPT) individuals, percent of a dominant taxon, percent taxa abundance without Chironomidae, Shannon’s diversity index, percent gatherer individuals, ratio of filterers and scrapers, and the Korean saprobic index. Our multimetric index successfully distinguished reference from impaired conditions. A scoring system was established for each core metric using its quartile range and response to anthropogenic disturbances. The multimetric index was classified by aggregating the individual metric ..scores and the value range was quadrisected to provide a narrative criterion (Poor, Fair, Good and Excellent) to describe the biological integrity of the streams in the study. A validation procedure showed that the index is an effective method for evaluating stream conditions, and thus is appropriate for use in future studies measuring the long-term status of streams, and the effectiveness of restoration methods.

## 1. Introduction

Streams and rivers are among the most threatened ecosystems worldwide, affected by increasing water demands by the human population and a variety of development pressures [1,2,3]. Such anthropogenic activities generally alter hydrology, water quality, physical in-stream and riparian environments, and aquatic biota, consequently leading to the overall ecological integrity of aquatic ecosystems. The practices of restoring ecological integrity, therefore, are going to be major tools for mitigating, arresting and reversing the adverse effects human activity has had on the aquatic system. Particularly, biological assessments have been increasingly recognized to be among the most underpinning procedure of the remediation practice [4,5,6,7].

Biological assessments intend to characterize the current status of stream ecosystems by monitoring changes in the aquatic communities associated with anthropogenic disturbance. Since the so-called “Saprobien system” [8,9], a large number of studies of aquatic communities have been undertaken to establish the effective methods for the assessment of stream water quality. Initially, assessments of the status of streams involved simple qualitative systems based only on the absence or presence of indicator species according to the gradient of environmental factors [10]. However, the indicator species concept is an inadequate measure of overall ecological integrity because the cause–effect relationships of indicator organisms are not fully established, and are often confusing [11,12]. Thus, alternative bio-assessment approaches, such as multimetric indices, have been developed to reflect all types of degradation and cumulative impacts at the ecosystem level. A multimetric index is composed of several metrics associated with biological attributes (*i.e.*, taxa richness, composition, pollution tolerance and trophic structure) that change in a predictable fashion with increasing anthropogenic disturbance [13]. This approach had the potential for broad use in the assessment of stream ecosystems because it involved various types of measurement and provided comprehensive comparative information relative to pre-determined criteria derived from non-impacted reference conditions [14,15,16].

Since the first multimetric method which was developed based on fish communities for assessing the biological integrity of streams [14], various types of multimetric indices have been subsequently proposed in other aquatic habitats and also in terrestrial environments, using different biological communities including periphyton [17], benthic macroinvertebrates [13,16,18], fishes [19,20], plants [21], birds [22], amphibians [23] and terrestrial invertebrates [24]. Of the organisms used as biological indicators in research and monitoring programs, benthic macroinvertebrates are the most commonly used assemblages worldwide [25]. Characteristics including high diversity, a relatively long life-span, bottom-dwelling life style, and sensitivity on environmental disturbance make them suitable for assessing the ecological status of lotic ecosystems [26,27]. As biological indicators, they can provide insights into the current and past conditions of a water body and integrate the effects of cumulative stressors [4,28].

In developing multimetric indices using benthic macroinvertebrates in various stream environments worldwide, a great number of metrics (up to 237 [29]) have been examined and accordingly evaluated in streams [4,16,29,30,31,32,33,34]. However, the practical number of metrics and their properties that have ended up being included in the developed indices vary among different multimetric indices. This indicates the possibility of metric variability responding to different environmental gradients in specific geographic regions. Moreover, robustness of selected metrics requires relatively long term validation for practical use, not only because the metric data used in the index development do not reflect the range of long term changes, but also because the streams targeted for the assessment are facing various environmental pressures. Therefore, biological indices, including multimetric indices developed in particular geographic regions or environments, are frequently used elsewhere [34]. Such metrics may be less useful when applied in regions other than that where the species-environment relationships were originally assessed [17,35].

In Korea, a variety of attempts has been made over the past 20 years to identify and assess the degree of impairment of stream ecosystems using benthic macroinvertebrates. However, most of these have used assessment methods developed for particular geographic regions in other countries, or have been focused largely on relationships to chemical variables. A number of biological methods specific to certain stream environments have been based on the indicator species concept. These include the Total Biotic Score (TBS) [36] and its revised version, the Korean Saprobic Index (KSI) [37], which are quantitative indices based on the method of Zelinka-Marvan [38]; the Group Pollution Index (GPI) [39] and the Ecological Score of Benthic macroinvertebrate community (ESB) [40], which are cost-effective qualitative methods. These methods only partially consider water quality, and their results vary with the sampling methods used. Thus, an integrated assessment method is necessary to provide information on biological integrity, and to enable measurement of the long-term health status of streams and the effectiveness of various remediation methods. 

The purpose of this study was to develop a multimetric index (the Korean Benthic macroinvertebrate Index for Biological Integrity—KB-IBI) using benthic macroinvertebrate communities for the assessment of the biological integrity in Korean streams, and potentially for application to streams in other countries. 

## 2. Materials and Methods

### 2.1. Study Area

South Korea is located between 37°00'N and 127°30'N, and has an area of approximately 100,033 km^2^ that encompasses the southern half of the Korean peninsula. The annual precipitation is 1,308 mm, but there is substantial variation among seasons [41]. Korean streams are affected by flooding as a consequence of high levels of precipitation during the summer monsoon period, but in other seasons only maintain base flow or may not flow at all because of drought conditions. Detailed information about the major rivers and their watershed conditions can be found in the related studies [42,43]. 

**Figure 1:**
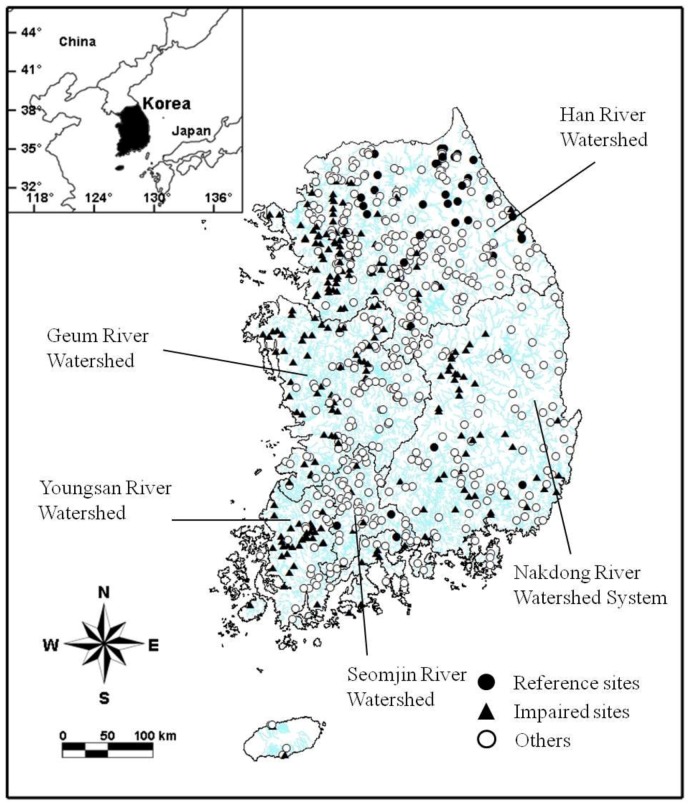
Spatial distribution of study sites assigned to each of the three status categories (reference, impaired, others), according to site classification criteria.

The National Aquatic Ecosystem Monitoring Program (NAEMP) has undertaken a bi-annual evaluation of the ecosystem health status of Korean streams [44]. This program included a total of 720 sampling sites in 388 streams and rivers in 2009, and included more than five major river systems throughout the country (Figure 1). The number of sampling sites will be extended to 1,200 until 2015, when they will be included under regulation in the national biological monitoring network [5]. While the waterways involved range from small mountainous streams to large rivers, most sampling sites were in wadeable streams. The largest numbers of sampling sites were in the Han River watershed (n = 320), followed by the Nakdong and Geum river watersheds (n = 130 each), the Youngsan River watershed (n = 76), and the Seomjin River watershed (n = 64). This large-scale national monitoring program included the majority of stream types in Korea, providing the basis for the development of a multimetric index. Physico-chemical and biological data were compiled from the NAEMP surveys conducted during May 2009.

Biological data from a total of ninety-six sampling events within two National Parks were included to complement the establishment of reference sites because the NAEMP was mostly concentrated on middle reach and lowland streams for monitoring and restoration activities. These streams ranged from second to fourth stream order, and are representative of reference conditions because they are well preserved by strict natural resources conservation laws [45,46]. Field samplings were performed at the same period of NAEMP survey under base flow conditions. This study used such data only for the procedures of metric selection and index development due to the lack of physico-chemical information.

### 2.2. Measurement of Environmental Variables

Environmental parameters were measured at each study site. Regional variables included altitude and land use type for watershed characteristics. Altitude was determined using a digital elevation model (DEM), and for each sampling site a topographic map (1:50,000) was used to qualitatively characterize the watershed according to the proportions of forest, agriculture and urban land use categories. Hydraulic and physical properties at the local scale were measured at each site, including: (i) average current velocity, measured at riffles or gliding runs using a current meter, or calculated by the Craig method [47]; (ii) the percent substrate composition, which was visually estimated with respect to fine (<2 mm) and coarse (≥2 mm) particle size classes; and (iii) habitat-riparian quality, using a habitat indexing system (HIS) comprising ten component metrics that reflect channel development, lateral and longitudinal connectivity, bank stability, substrate condition and riparian land use [48].

Water quality parameters including pH, dissolved oxygen (DO), turbidity and electrical conductivity (EC) were measured using a portable multi-probe meter (YSI 6920, YSI Inc., Yellow Springs, OH, USA or Horiba U-22XD, Kyoto, Japan) at the center of each sampling site. In addition, 2 L of stream water was sampled at each site in sterilized plastic bottles that were transported on ice to the laboratory for measurement of biochemical oxygen demand (BOD), total nitrogen (TN) and total phosphorus (TP), following standard methods [49].

### 2.3. Benthic Macroinvertebrate Sampling

A Surber sampler (30 × 30 cm; net mesh size 1 mm) was used to quantitatively collect benthic macroinvertebrates from the center of the channel at each site. Three replicate samples, randomly taken at each riffle or gliding run within a 100 m reach, were pooled in a 500 mL plastic bottle to which 80% ethanol was immediately added. The organisms in these samples were hand-separated from detritus and inorganic materials in the laboratory. Subsampling was undertaken only for dominant species (e.g., Oligochaeta, Ephemerellidae, Chironomidae and Hydropsychidae) where large numbers of specimens were present in each sample. Macroinvertebrate species were identified to the lowest possible taxonomic level (usually species or genus) by light microscopy using available keys [50,51,52,53]. However, the identification of several taxonomic groups belonging to the Annelida, Coleoptera and Diptera was restricted to the order or family level because of limited information on their systematics. All individuals were counted following identification. Macroinvertebrates were also classified into habit and functional feeding groups [51,54].

### 2.4. Site Classification

The reference sites for developing a multimetric index were established in areas that were as minimally impaired as possible [13,29,55], based on the site classification criteria. Reference sites had to satisfy minimal requirements concerning development and land use, and comprise >60% forest and <30% and 20% of agricultural and urban areas, respectively. For the requirement for excellent or good water quality status, four standards of stream water chemical quality set by the Ministry of Environment, Korea [44] were used as a reference, including biochemical oxygen demand ≤2 mg/L, total nitrogen <3 mg/L, total phosphorus <0.04 mg/L, and turbidity <10 NTU. The physical habitat conditions of reference sites included high heterogeneity in substrate composition (>60% coarse particles) and optimal or good HIS scores (>31). The chosen reference sites met all these criteria. A sampling site was classified as having impaired conditions if there was: a high degree of development pressure (<20% forested area); organic pollution (producing >8 mg/L BOD); nutrient enrichment (>6 mg/L in TN and >0.30 mg/L in TP), high turbidity (>50 NTU) and deterioration in habitat quality (>70% fine particles and a HIS score ≤ 20). Sites that were not classified as either reference or impaired were classed as “others”. The statistical differences between reference and impaired sites for the various environmental parameters were assessed using the non-parametric Mann-Whitney test.

### 2.5. Metric Selection and Index Development

Development of the macroinvertebrate-based multimetric index generally followed the procedure of Barbour *et al.* [13]. Thirty-four candidate metrics were screened and examined; these included properties of richness, composition, trophic/habit status and tolerance measures, which were derived from previous studies [13,16,56,57] (Table 1). Comparison of the metric values between reference and impaired streams was undertaken with the aim of selecting the most appropriate metrics for Korean streams. Metric selection was performed using a stepwise process involving assessment of their variation, redundancy and sensitivity. 

**Table 1:** Definition of 34 candidate metrics and their expected responses to increasing anthropogenic disturbance.

Metric	Definition	Expected
		Response
**Richness measures**		
Number of taxa	Number of species collected in the sample	Decrease
Number of EPT taxa	Number of taxa in the orders Ephemeroptera, Plecoptera, and Trichoptera	Decrease
Number of Ephemeroptera	Number of mayfly (Ephemeroptera) nymphs	Decrease
Number of Plecoptera	Number of stonefly (Plecoptera) nymphs	Decrease
Number ofTrichoptera	Number of caddisfly (Trichoptera) larvae	Decrease
**Composition measures**		
% Dominant taxon	Percent of individuals in the most abundant species	Increase
% Oligochaeta	Percent of individuals in aquatic worms	Increase
% EPT taxa	Percent of individuals in the insect orders Ephemeroptera, Plecoptera, and Trichoptera	Decrease
% Ephemeroptera	Percent of individuals in mayfly nymphs	Decrease
% Plecoptera	Percent of individuals in stonefly nymphs	Decrease
% Trichoptera	Percent of individuals in caddisfly larvae	Decrease
% Chironomidae	Percent of individuals in chironomid midge larvae	Increase
**Composition measures**		
% Taxa abundance without Chironomidae	Percent of individuals in taxa abundance without chironomid midge larvae	Decrease
% Non-insects and Chironomidae	Percent of individuals in non-insects and chironomid midge larvae	Increase
% Non-insects	Percent of individuals in non-insects	Increase
Total density	Total abundance converted to number per square meter	Variable
Ratio of EPT to Chironomidae	Ratio of pollution sensitive EPT taxa to pollution tolerant chironomid midge larvae	Decrease
McNaughton’s dominance index	McNaughton’s dominance index [58]	Increase
Shannon’s diversity index	Shannon’s diversity index [59]	Decrease
Margalef’s richness index	Value of Margalef’s species richness index [60]	Decrease
Pielou’s evenness index	Value of Pielou’s evenness index [61]	Decrease
**Trophic/habit measures**		
% Shredders	Percent of individuals in the shredder functional feeding group	Decrease
% Scrapers	Percent of individuals in the scraper functional feeding group	Decrease
% Filterers	Percent of individuals in the collector-filterer functional feeding group	Decrease
% Gatherers	Percent of individuals in the collector-gatherer functional feeding group	Variable
% Predators	Percent of individuals in the predator functional feeding group	Variable
Ratio of filterers and scrapers	Ratio of collector-filterers and scrapers to total density	Decrease
Ratio of scrapers to filterers	Ratio of scrapers to collector-filterer functional feeding group	Decrease
Number of clingers	Number of the clinger functional habit group	Decrease
Number of clingers and sprawler	Number of the clinger and sprawler functional habit groups	Decrease
% Clingers	Percent of individuals in the clinger functional habit group	Decrease
% Clingers and sprawlers	Percent of individuals in the clinger and sprawler functional habit groups	Decrease
**Tolerance measures**		
ESB	Sum of assigned ecological scores for each occurring species in the macroinvertebrate assemblage [40,62]	Decrease
KSI	Value of Korean saprobic index [37]	Increase
**Composition measures**		
% Taxa abundance without Chironomidae	Percent of individuals in taxa abundance without chironomid midge larvae	Decrease
% Non-insects and Chironomidae	Percent of individuals in non-insects and chironomid midge larvae	Increase
% Non-insects	Percent of individuals in non-insects	Increase
Total density	Total abundance converted to number per square meter	Variable
Ratio of EPT to Chironomidae	Ratio of pollution sensitive EPT taxa to pollution tolerant chironomid midge larvae	Decrease
McNaughton’s dominance index	McNaughton’s dominance index [58]	Increase
Shannon’s diversity index	Shannon’s diversity index [59]	Decrease
Margalef’s richness index	Value of Margalef’s species richness index [60]	Decrease
Pielou’s evenness index	Value of Pielou’s evenness index [61]	Decrease
**Trophic/habit measures**		
% Shredders	Percent of individuals in the shredder functional feeding group	Decrease
% Scrapers	Percent of individuals in the scraper functional feeding group	Decrease
% Filterers	Percent of individuals in the collector-filterer functional feeding group	Decrease
% Gatherers	Percent of individuals in the collector-gatherer functional feeding group	Variable
% Predators	Percent of individuals in the predator functional feeding group	Variable
Ratio of filterers and scrapers	Ratio of collector-filterers and scrapers to total density	Decrease
Ratio of scrapers to filterers	Ratio of scrapers to collector-filterer functional feeding group	Decrease
Number of clingers	Number of the clinger functional habit group	Decrease
Number of clingers and sprawler	Number of the clinger and sprawler functional habit groups	Decrease
% Clingers	Percent of individuals in the clinger functional habit group	Decrease
% Clingers and sprawlers	Percent of individuals in the clinger and sprawler functional habit groups	Decrease
**Tolerance measures**		
ESB	Sum of assigned ecological scores for each occurring species in the macroinvertebrate assemblage [40,62]	Decrease
KSI	Value of Korean saprobic index [37]	Increase

EPT, Ephemeroptera–Plecoptera–Trichoptera; ESB, Ecological Score of Benthic macroinvertebrate community; KSI, Korean Saprobic Index.

Metric suitability was initially assessed for discrimination of reference from impaired conditions. We discarded metrics with low values and large variability from the reference site group, because of their poor discrimination ability. For all combinations of the metrics found to be suitable according to this criterion, a redundancy test was performed to detect redundant metrics in the index, using Pearson’s correlation analysis. High correlation coefficients (*r* > 0.80, *p* < 0.05) were interpreted as indicating redundant metrics, and in these cases only one metric was retained for further assessment in the index development procedure.

Box-and-whisker plots, which enabled visualization of variations in the metric ranges, were used to estimate the ability of metrics to discriminate reference and impaired sites. The discriminatory power of each remaining metric was determined according to the degree of overlap of medians and interquartile ranges [13]. Metrics with no overlap of interquartile ranges were considered to have good discriminatory power for both reference and impaired sites.

A multimetric index should include metrics reflecting ecological characteristics, and be able to indicate potential stressor–specific relationships [16]. Thus, the responsiveness of each remaining metric to anthropogenic disturbance was evaluated using Pearson’s correlation analysis. Those metrics that correlated with at least one of the environmental variables at *p* < 0.01 were accepted.

The final multimetric index was constructed from a combination of the core metrics selected through the metric selection procedure. A scoring system of 1, 3 or 5 points was adopted using threshold values (the minimum, the 25th percentile, the 75th percentile, and the maximum) for each component metric, according to its response to environmental degradation [13,55,56]. The index value for each sampling site was obtained by aggregating the individual core metric scores. The index range was then quadrisected to generate four classes: Class A (Excellent), which indicated the site was comparable in condition to the reference biological conditions; Class B (Good) indicating slight disturbance; Class C (Fair), indicating moderate disturbance; and Class D (Poor), indicating severely disturbed biological integrity. 

### 2.6. Index Validation

The sensitivity of the multimetric index was determined by assessing whether there was clear discrimination among the classified site groups (reference, impaired and others) using box-and-whisker plots. Pearson’s correlation analysis was also used to identify relationships between the index scores and environmental variables. Principle components analysis (PCA) was used to examine the responsiveness of the multimetric index to environmental variables, as illuminating the distribution of sites in the ordination space and statistical correlations. PCA was performed using PC-ORD software (version 4.25) [63].

## 3. Results

### 3.1. Site Classification and Environmental Characteristics

The site classification screening procedure identified 135 reference sites (39 sites from NAEMP database and 96 from two National Parks), most of which were distributed over the Han River watershed, and 236 impaired sites. There were significant differences in environmental parameters between the reference and impaired sites except for pH, DO and turbidity (*p *< 0.05) (Table 2). 

**Table 2:** Summary statistics of environmental variables for reference and impaired sites, showing the average (standard deviation) and range (minimum and maximum values) for each variable.

Variable ^1^	Reference site (n = 39)			Impaired site (n = 236)		*P*
	Average	Range		Average	Range	
pH	8.1 (0.7)	7.0–9.5		8.0 (0.8)	6.6–11.1	0.481
DO (mg L^−1^)	9.9 (1.7)	6.0–12.6		9.5 (3.0)	2.4–17.3	0.536
BOD (mg L^−1^)	1.1 (0.5)	0.5–2.7		4.9 (4.1)	0.3–37.5	<0.001^*^
EC (mS m^−1^)	122.0 (136.7)	31–818		1,172.5 (4,637.5)	82.4–40,600.0	<0.001^*^
TN (mg L^−1^)	1.51 (0.62)	0.38–2.96		4.76 (3.81)	0.32–27.71	<0.001^*^
TP (mg L^−1^)	0.02 (0.02)	0.00–0.11		0.30 (0.48)	0.00–5.59	<0.001^*^
Turbidity (NTU)	5.7 (3.8)	0–11		15.3 (35.2)	0.0–400.0	0.080
Altitude (m)	285.6 (181.5)	33–718		37.7 (36.8)	0.0–233.0	<0.001^*^
% Urban	5.9 (4.8)	0–15		36.4 (36.6)	0.0–100	<0.001^*^
% Agriculture	10.5 (11.2)	0–30		40.4 (35.1)	0.0–100	<0.001^*^
% Forest	83.3 (12.9)	60.0–100.0		15.9 (23.4)	0.0–100	<0.001^*^
Velocity (cm sec^−1^)	65.7 (25.4)	18.7–105.7		24.5 (22.6)	0.0–95.8	<0.001^*^
% Fine	11.2 (9.1)	0–30		67.2 (31.6)	0.0–100	<0.001^*^
% Coarse	88.8 (9.1)	70–100		32.4 (31.3)	0.0–100	<0.001^*^
HIS	41.5 (4.2)	31–50		32.5 (7.0)	0.0–50	<0.001^*^

^1 ^Abbreviations for environmental variables are; DO, dissolved oxygen; BOD, biochemical oxygen; EC, electrical conductivity; TN, total nitrogen; TP, total phosphorus; HIS, habitat indexing system.

* Reference and impaired sites showed significant difference at *p *< 0.01 (Mann-Whitney test).

Consistent with the physico-chemical criteria for site classification, the reference sites were primarily distributed in forested mountainous areas (average forest cover and height a.s.l. = 83.3% and 285.6 m, respectively), and had excellent water quality. In contrast, the impaired sites had fair to poor water quality, and were characterized by high levels of urban development and agricultural land uses, although these parameters varied considerably depending on geographical location. The HIS scores, which indicated habitat and riparian quality, were also lower for sites in the impaired group (average 32.5) than the reference group (41.5).

### 3.2. Metric Evaluation

#### 3.2.1. Metric Variability

Thirteen of the initial 34 candidate metrics did not meet the first criterion because their values were too low or variable to enable detection of degradation in habitat quality (Table 3). The metrics discarded because of low values were the number of Plecoptera, and the metrics for percent individuals of Oligochaeta, Plecoptera, non-insects, shredders, filterers and predators. The metrics rejected because of their high degree of variation were percent Chironomidae individuals, percent non-insects and Chironomidae individuals, total density, the ratio of EPT individuals to Chironomidae, percent clinger and sprawler individuals, and the ratio of scrapers to filters. 

**Table 3:** Summary statistics of the initial 34 candidate metrics at reference sites, showing the interquartile values (25th, median, and 75th percentiles). Those metrics in bold were the eight core metrics included in the multimetric index.

Metric ^1^	Interquartile values			Reason for rejection
	25th percentile	Median	75th percentile	
**Number of taxa**	24	30	37	
Number of EPT taxa	18	22	28	Redundant
Number of Ephemeroptera	9	11	13	Redundant
Number of Plecoptera	2	5	7	Values low, variable
Number of Trichoptera	5	7	9	Redundant
**% Dominant taxon**	18.6	23.9	30.6	
% Oligochaeta	0.0	0.0	0.8	Values low
**% EPT**	72.3	76.9	81.5	
% Ephemeroptera	31.1	37.0	43.9	Redundant
% Plecoptera	10.2	15.4	23.2	Values low, variable
% Trichoptera	19.3	23.1	27.7	Redundant
% Chironomidae	5.6	10.6	17.0	Variable
**% Taxa abundance without Chironomidae**	83.0	89.4	94.4	
% Non-insects and Chironomidae	8.6	13.2	19.7	Variable
% Non-insects	0.5	1.8	4.0	Values low
Total density	860	1961	2689	Variable
Ratio of EPT to Chironomidae individuals	4	7	14	Variable
McNaughton’s dominance index	0.33	0.40	0.48	Redundant
**Shannon’s diversity index**	3.34	3.68	4.02	
Margalef’s species richness index	3.16	3.86	4.52	Redundant
Pielou’s evenness index	0.71	0.76	0.80	Redundant
% Shredders	1.1	3.4	10.4	Values low
% Scrapers	28.4	41.5	50.9	Redundant
% Filterers	1.8	5.0	10.4	Values low
**% Gatherers**	23.3	33.1	42.2	
% Predators	6.6	10.1	14.0	Values low
**Ratio of filterers and scrapers**	0.39	0.48	0.59	
Ratio of scrapers to filterers	3.3	7.1	16.0	Variable
Number of clingers	14	18	22	Redundant
Number of clingers and sprawlers	18	24	29	Redundant
% Clingers	51.0	61.7	73.8	Redundant
% Clingers and sprawlers	57.6	71.5	81.0	Variable
ESB	84	109	133	Redundant
**KSI**	0.16	0.21	0.33	

^1^ EPT; Ephemeroptera-Plecoptera-Trichoptera; ESB, Ecological Score of Benthic macroinvertebrate community; KSI, Korean Saprobic Index.

#### 3.2.2. Metric Redundancy

Based on Pearson’s correlation analysis (*r* > 0.80), of the remaining 21 metrics there were strong linear correlations among number of taxa, number of EPT taxa, number of clingers, number of clingers and sprawlers, Margalef’s species richness index, and ESB. Only number of taxa was retained because of its sensitivity and wide use. The percent dominant taxon metric was also redundant with McNaughton’s dominance index (*r* = 0.930, *p* < 0.01). Among trophic and habit metrics, percent clinger individuals and percent scraper individuals were eliminated because of redundancy with the ratio of filterers and scrapers (*r* = 0.815 and *r* = 0.825, respectively). 

When particular taxonomic groups were duplicated among the remaining metrics, more powerful measures for differentiating reference and impaired sites were chosen to provide a more operational and simple index. Using this rationale, the metric percent of EPT individuals was chosen instead of either percent Ephemeroptera or Trichopteran individuals because of its greater sensitivity and its common use worldwide [4,64,65]. Pielou’s evenness metric was removed because Shannon’s diversity index incorporated both species richness and evenness [13,26]. 

#### 3.2.3. Discriminatory Power of Metric Sensitivity

**Figure 2:**
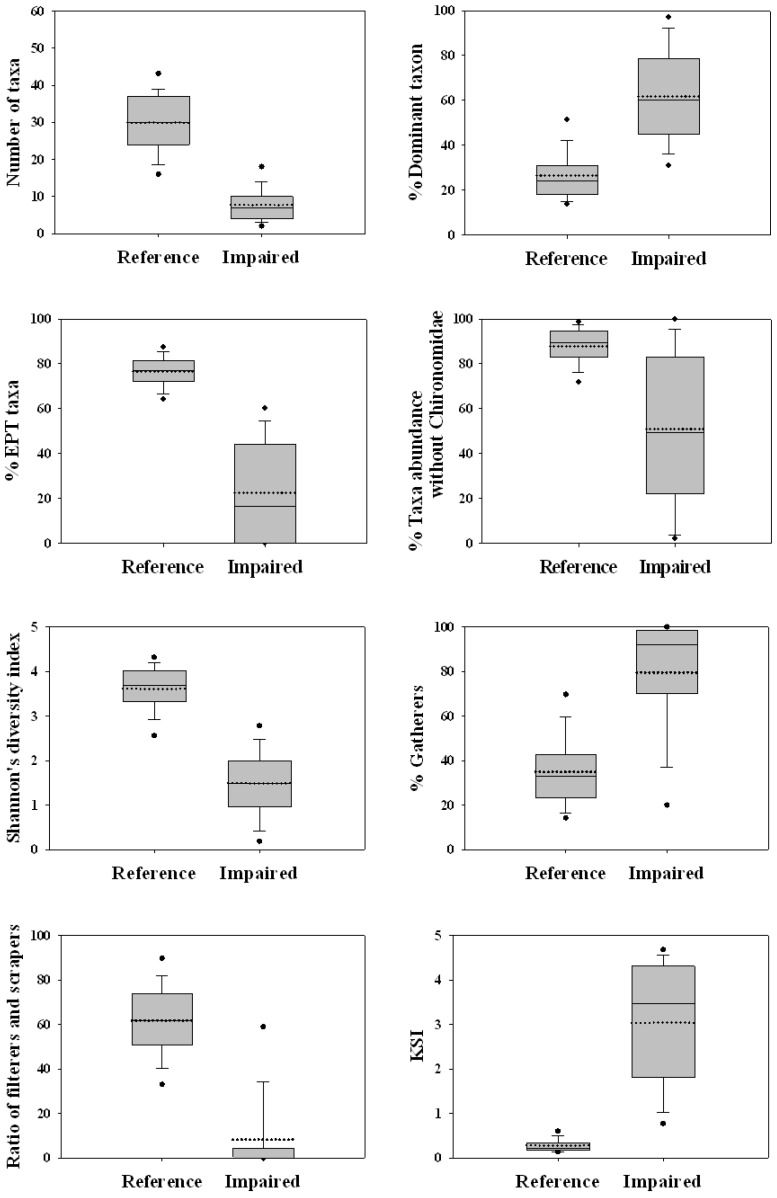
Comparison of core metric values between the reference and impaired site groups. Boxes represent interquartile ranges (25th–75th percentiles). Closed circles at the bottom and top of each box indicate the 5th and 95th percentiles, respectively. The mean (horizontal dotted line), median (horizontal solid line), and standard deviation (error bar) are shown in the box plots.

The remaining eight metrics of the initial 34 candidates met the first (variability) and second (redundancy) criteria, and all clearly distinguished reference and impaired sites (Figure 2). None of the eight metrics showed partial or considerable interquartile overlaps. Furthermore, the interquartile ranges of the reference site group were much narrower than those of the impaired group.

#### 3.2.4. Relationship between Metrics and Environmental Variables

All eight metrics were significantly correlated with environmental variables (*p *< 0.01). The correlation coefficients for physical variables were much higher than those for chemical variables (Table 4). In particular, altitude and percent forest cover were the most significant attributes affecting metric values. Three metrics, percent dominant taxon, percent gatherer individuals and KSI had positive relationships with water quality parameters, whereas the others were negatively correlated. The core metrics selected for the multimetric index were number of taxa, percent EPT individuals, percent dominant taxon, percent taxa abundance without Chironomidae, Shannon’s diversity index, percent gatherer individuals, ratio of filterers and scrapers, and the KSI.

**Table 4:** Pearson’s correlation coefficients between the eight core metrics, the multimetric KB-IBI, and environmental variables (n = 275). ** *p* < 0.01, * *p* < 0.05.

Metric ^1^	Environmental variable ^2^									
	BOD	EC	TN	TP	Turbidity	Altitude	%Forest	Velocity	%Coarse	HIS
Number of taxa	−0.136 ^*^	−0.096	−0.166^ **^	−0.122^ *^	−0.101	0.543^ **^	0.525 ^**^	0.329 ^**^	0.460 ^**^	0.296 ^**^
% Dominant taxon	0.204 ^**^	0.001	0.150 ^*^	0.151 ^*^	0.070	−0.334 ^**^	−0.394 ^**^	−0.250 ^**^	−0.333 ^**^	−0.205 ^**^
% EPT	−0.160 ^**^	−0.075	−0.153 ^*^	−0.173 ^**^	−0.041	0.475 ^**^	0.474 ^**^	0.339 ^**^	0.386 ^**^	0.205 ^**^
% Total abundance without Chironomidae	−0.080	−0.112	−0.052	−0.037	−0.038	0.200 ^**^	0.176 ^**^	0.244 ^**^	0.199 ^**^	−0.013
Shannon’s diversity index	−0.213 ^**^	−0.048	−0.178 ^**^	−0.147 ^*^	−0.103	0.480 ^**^	0.532 ^**^	0.333 ^**^	0.448 ^**^	0.307 ^**^
% Gatherers	0.195 ^**^	0.081	0.152^*^	0.089	0.055	−0.300 ^**^	−0.330 ^**^	−0.228 ^**^	−0.316 ^**^	−0.252 ^**^
Ratio of filterers and scrapers	−0.225 ^**^	−0.089	−0.209 ^**^	−0.167 ^**^	−0.072	0.399 ^**^	0.465 ^**^	0.285 ^**^	0.328 ^**^	0.311 ^**^
KSI	0.230 ^**^	0.136 ^*^	0.208 ^**^	0.146 ^*^	0.075	−0.415 ^**^	−0.435 ^**^	−0.256 ^**^	−0.286 ^**^	−0.316 ^**^
KB−IBI	−0.370 ^**^	−0.084 ^*^	−0.397 ^**^	−0.292 ^**^	−0.144 ^**^	0.387 ^**^	0.444 ^**^	0.465 ^**^	0.492 ^**^	0.432 ^**^

^1^ EPT, Ephemeroptera–Plecoptera–Trichoptera; KSI, Korean Saprobic Index; KB−IBI, Korean Benthic macroinvertebrate Index of Biological Integrity.

^2^ Abbreviations for environmental variables are; BOD, biochemical oxygen; EC, electrical conductivity; TN, total nitrogen; TP, total phosphorus; HIS, habitat indexing system.

### 3.3. Development of the Multimetric Index

The scoring criteria for each metric were effectively determined using threshold values from reference sites (Table 5). The multimetric index was calculated by aggregating the scores of each of the eight metrics. The possible index values ranged from 8 to 38; this was derived by summing the minimum and maximum scores for each metric. The range of the multimetric index was quadrisected to define four classes of biological integrity (Table 6); excellent condition (scores of 32–38) equivalent to the reference condition; good (24–31), having slightly impaired biological condition; fair (16–23), having moderate impairment; and poor (8–15), which indicated severe impairment and unsatisfactory biological integrity.

**Table 5:** Descriptive statistics (minimum, 25th percentile, median, 75th percentile, and maximum) for the eight core metrics incorporated into the multimetric KB-IBI, and the scoring criteria for each. The KSI metric showed relatively weak discriminatory power because of low values, and the scoring criteria were therefore reduced two scores (3 and 1) to reduce its effect on the KB-IBI.

Metric ^1^	Descriptive statistics						Scoring criteria		
	Min	25%	Median	75%	Max		5	3	1
Number of taxa	13	24	30	37	53		≥24	13–23	≤12
% Dominant taxon	10.4	18.6	23.9	30.6	70.3		≤31.0	31.1–70.0	≥70.1
% EPT individuals	57.1	72.3	76.9	81.5	90.9		≥72.0	57.0–71.9	≤56.9
% Taxa abundance without Chironomidae	41.3	83.0	89.4	94.4	100.0		≥83.0	41.0–82.9	≤40.9
Shannon’s diversity index	2.02	3.34	3.68	4.02	4.70		≥3.34	2.00–3.33	≤1.99
% Gatherer individuals	7.5	23.3	33.1	42.2	75.4		≤42.0	42.1–75.0	≥75.1
Ratio of filterer and scraper to total density	0.10	0.39	0.48	0.59	0.84		≥0.39	0.10–0.38	≤0.09
KSI	0.11	0.16	0.21	0.33	1.53		-	≤1.53	>1.53

^1 ^EPT, Ephemeroptera–Plecoptera–Trichoptera; KSI, Korean Saprobic Index.

**Table 6:** KB-IBI guideline for classifying biological integrity of stream ecosystems.

KB-IBI score	Integrity class	Environmental quality and biological characteristics
32–38	Excellent	No evidence or minor problems of anthropogenic disturbance with optimal habitat quality Desirable biological integrity comparable to the reference condition High species diversity and dominance by pollution-intolerant taxa Rhithrophilic species dominated with heterogeneous coarse substrates
24–31	Good	Low levels of disturbance impacts on communities and their habitats Trivial increases in abundance of non-insect taxa Slight increases in pollution-tolerant taxa but intolerant taxa still dominated Species diversity somewhat below expectation
16–23	Fair	Significant impacts of anthropogenic disturbance on assemblages and habitat quality Noticeable decreases in taxa richness and abundance Evident increases in proportion of non-insect individuals and dominance of a dominant taxon Moderate changes in assemblages from pollution-intolerant to pollution-tolerant taxa
8–15	Poor	Severe disturbance and deterioration in habitat quality Severely impaired and unsatisfactory biological integrity Only a few pollution-tolerant taxa present in large numbers Potamal species dominated with homogeneous fine-sized substrates

### 3.4. Validation of the Multimetric Index

**Figure 3:**
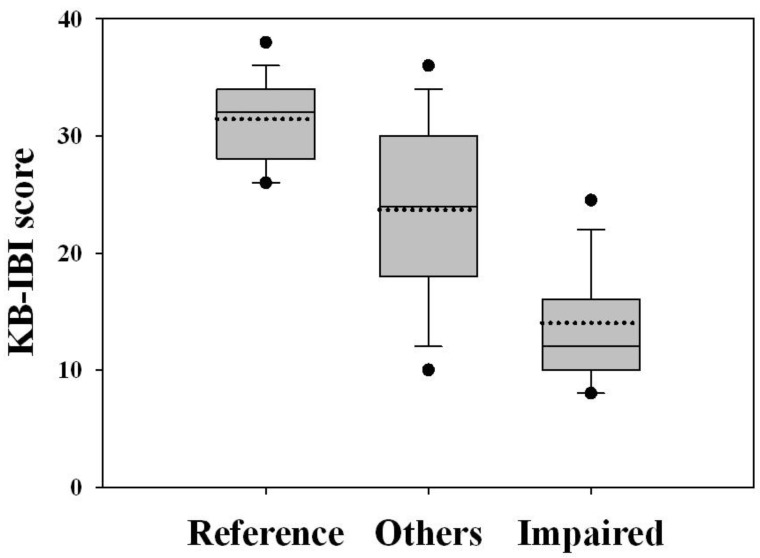
Comparison of the KB-IBI scores among reference, impaired and other sites. See Figure 2 for the description of the box plots.

The multimetric index was able to discriminate the reference and impaired site groups (Figure 3). The application of this index classified 112 sites of the reference group (83.0%) as having excellent biological integrity, which was consistent with the initial site classification, and the remainder of the reference sites was classified as being of good condition. The majority of sites initially considered to be impaired were categorized as being in fair or poor condition (91.5%).

The multimetric index showed good responsiveness to all environmental factors, as evidenced by significant statistical correlations (Table 4). Factors indicating anthropogenic activities, including percent urban and agricultural land uses, water quality parameters and percent fine substrates were negatively correlated with the index (*p* < 0.01), reflecting the impacts of human disturbance on the biological integrity of the streams concerned. In contrast, there were positive correlations with percent coarse substrates (*r* = 0.492, *p* < 0.01), current velocity (*r* = 0.465, *p* < 0.01), percent forest (*r* = 0.444, *p* < 0.01), and altitude (*r* = 0.387, *p* < 0.01).

**Figure 4:**
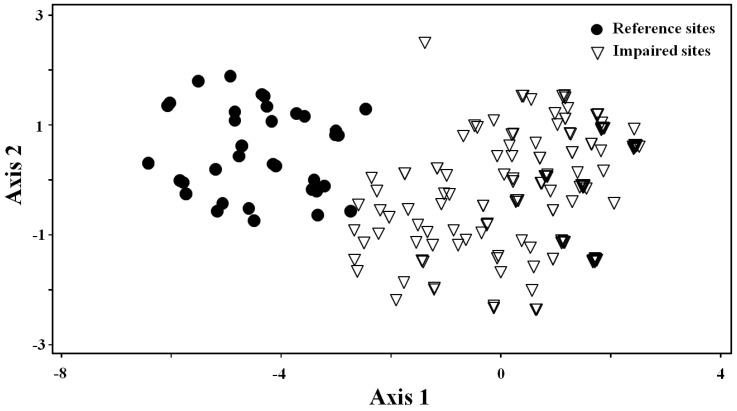
Ordering of reference and impaired sites by the first two principle components (PC1 and PC2) based on eight core metrics.

The first three PCA axes explained 80.8% of the inter-site variance, with eigenvalues >1. The reference sites grouped closely together on the left side of the PC1 axis, while the impaired sites were scattered to the right side (Figure 4). The PCA result also indicated a predictable pattern of site distribution in relation to environmental variables. Among the three axes, PC1 was positively correlated with human land uses and nutrients, and negatively correlated with altitude and physical instream quality (Table 7). These results indicated good responsiveness of the multimetric index to variables of anthropogenic disturbance along the PC1 axis. However, both PC2 and PC3 showed weak correlations with environmental variables.

**Table 7:** Pearson correlation coefficients between first three axes of the PCA and the environmental variables measured. ** *p *< 0.01, * *p *< 0.05.

Variable ^1^	PC1	PC2	PC3
pH	–0.048	0.100	–0.053
DO	–0.146^*^	0.017	–0.039
BOD	0.367^**^	–0.139^*^	0.047
EC	0.041	–0.120^*^	0.032
TN	0.379^**^	0.012	0.042
TP	0.240^**^	–0.065	0.035
Turbidity	0.074	–0.109	0.008
Altitude	–0.634^**^	0.097	–0.179^**^
% City	0.350^**^	–0.069	0.131^*^
% Agriculture	0.203^**^	0.072	–0.011
% Forest	–0.700^**^	0.040	–0.108
Velocity	–0.467^**^	0.106	–0.142^*^
% Fine	0.504^**^	–0.039	0.042
% Coarse	–0.504^**^	0.039	–0.042
HIS	–0.408^**^	–0.028	–0.111

^1 ^Abbreviations for environmental variables are; DO, dissolved oxygen; BOD, biochemical oxygen; EC, electrical conductivity; TN, total nitrogen; TP, total phosphorus; HIS, habitat indexing system.

## 4. Discussion

### 4.1. Significance of a Multimetric Index

Advances in human standards of living have involved greater use of water resources, which have at the same time been affected by major modifications of aquatic environments through industrialization, urbanization, deforestation, channelization, dam construction and dredging activities. Such anthropogenic disturbances have adversely influenced the ecological integrity of stream ecosystems, degrading both their physico-chemical and biological integrity. Biological monitoring based on various aquatic biota may be more effective than measuring water chemistry alone, because the organisms integrate the chemical and physical properties of streams over time, which could be overlooked by one-time water chemistry sampling [66].

The development of biological assessment methods using benthic macroinvertebrates has a history of approximately 100 years. Because of their wide distribution and distinct ecology, the assessment of stream macroinvertebrate communities has become a well-established method used by various countries to monitor waterways (e.g., references in [6]). Past approaches to monitoring and evaluating river/stream ecosystems in Korea have been largely limited to water chemistry (particularly BOD), which are inadequate for interpreting stream health/biotic integrity [11,13,40,67].

Not until early in the 21st century (2006) was there a major shift of water management policy concerning stream health and ecological restoration of disturbed streams in Korea, following which various biological monitoring methods have been developed based on diatoms, macroinvertebrates and fish [5]. As a consequence, the KSI was developed using macroinvertebrate communities, based on their relation to BOD [37]. However, the KSI has limitations for use in integrated biological assessment because: (i) it considers only 100 commonly occurring indicator groups; (ii) it is uncertain whether these indicator groups are adequate for discriminating ecosystem health; (iii) the assessment results may be questionable for streams where the indicators do not occur; and (iv) it often distorts the assessment of streams that are characterized as having good water quality, but have physically deteriorated conditions. Korean streams and rivers have recently been subject to sudden changes resulting from nation-wide maintenance practices that are supposedly directed at restoration. However, these restoration practices are controversial because of their negative effects on overall stream ecosystem structure and function (*i.e.*, health), despite minimizing water quality problems [5]. Thus, the evaluation of stream ecosystem health demands more effective and integrated assessment methods to enable detection of deterioration in overall environmental quality, and to assess the effectiveness of remediation measures.

A multimetric index integrates information from a variety of ecological measures that respond to human influence in a predictable way [12,14], and offers the potential to provide biological criteria for stream restoration [56]. Therefore, the index developed in this study may be an effective tool for monitoring and assessing the biological integrity of Korean streams, and also provide a useful reference for other bio-assessment methods.

For example, the Ohio Environmental Protection Agency (OEPA) in the U.S. currently uses the invertebrate community index (ICI) as its principal tool for assessing streams in Ohio [68]. The ICI is a multimetric index that uses 10 measures of taxonomic composition and functional groups to evaluate stream health. Both the ICI and our index include taxonomic and functional group metrics, but our index includes aspects of community diversity (Shannon’s diversity index) and water quality relationships (KSI). The metric of percent tolerant organisms in the ICI provides information on indicator species. As pointed out in some previous works, including indicator species metrics in a multimetric index has limitations with respect to wide application, because indicator species often reflect either regional or local properties of community distribution and environmental gradients [69,70]. We believe that both indices have strengths at the regional level. Therefore, robustness in the assessment of stream health and related remediation programs needs to bring with the simultaneous consideration of multi-biota and full suite of chemical constituents [5,69].

### 4.2. Selection of Reference Sites

The selection of appropriate reference sites is a critical step in developing a multimetric index because this facilitates comparisons between reference and impaired sites [15]. Although reference sites should be in a non-impaired condition or have minimal human disturbance, there are difficulties in defining reference conditions because few stream ecosystems throughout Korea are in a pristine state, and there is a lack of historical records for non-impaired streams; this is largely the situation worldwide [29,71]. An eco-regionalization method addressing the homogeneity of physiographic characteristics would be a useful complement for site classification [13,72].

It was difficult to find reference sites for this study from the NAEMP database, despite the large number of sampling sites included. This was because site selection of NAEMP principally focused on the impaired tributaries and mainstreams with more concerns for restoration rather than non-impaired streams. Also, most of reference sites were located within the Han River watershed due to geographical bias of the sampling sites in the program (*i.e.*, Han River watershed included the largest number of sampling sites. See Figure 1). This is why we included additional 96 sampling occasions from two well-preserved National Parks to complement the reference sites establishment. Reference sites were effectively differentiated based on regional habitat characteristics, which represented stream environment properties and biological communities [15,73]. This was reinforced by considering three aspects of environmental properties in identifying reference sites: watershed parameters reflecting potential non-point pollution sources for streams nearby; water quality parameters indicating organic pollution and nutrient enrichment; and substrate heterogeneity supporting diversity and abundance of benthic macroinvertebrates. This process was appropriate in that suitable reference sites were chosen (Table 2).

### 4.3. Metric Evaluation

Metric selection was performed on the basis that impaired sites supported less taxa richness and abundance than reference sites, because of unsuitable habitat conditions resulting from anthropogenic disturbance [1,13]. Thirty-four candidate metrics were initially evaluated for the multimetric index in terms of their applicability and sensitivity. Most of these had been applied in other countries, but we identified only eight metrics as being applicable to Korean streams (Table 5). The metrics incorporated in our multimetric index effectively reflected the ambient physico-chemical conditions and human influence, and thus they were worth to be the components to discriminate each biological integrity class.

Taxa richness is one of the most reliable indicators in most multimetric indices, and shows good responsiveness to human disturbance [13,56]. Among the five candidate richness measures, “number of taxa” was sufficiently sensitive to differentiate reference and impaired sites; high species richness indicates undisturbed conditions and acceptable stream ecosystem health [4]. Both “the number of Plecoptera” and “the number of EPT taxa” metrics have been reported to be excellent indicators in biological assessments [26], but they were not included in this study because of weak discriminatory power and redundancy, respectively. The potential use of these two metrics is attributable to their sensitivity to perturbation and the fact that crucial environmental factors affect their spatial distribution [74,75]. The other metrics related to taxa richness showed the same trend as “number of taxa”.

The metrics related to the proportional abundance of taxon to the whole community are alternative measures of community balance [13]. Three metrics in our index were related to composition measures. The “percentage of a dominant taxon” metric is a measure of community diversity [56]. A high degree of dominance by few tolerant taxa implies lowered diversity and increased disturbance. This metric was also reported to be an excellent indicator for detecting metal contamination [16]. Another metric to discriminate human influences was “percentage of EPT individuals”, which included three individual taxa well known for their sensitivity to environmental degradation. However, use of EPT-related metrics requires a cautious use because of different tolerance levels along pollution gradients, which is dependent on the particular taxonomic groups involved [76]. Several families in the EPT taxa (e.g., Baetidae, Caenidae, Hydropsychidae) are capable of tolerating a broad range of disturbance, even in polluted streams [77,78,79]. Information on the systematics of chironomid larvae in Korea is rudimentary, despite the ecological importance of this group and its diversity and abundance [80]. Metrics associated with the Chironomidae were not included because this taxon often confuses the interpretation of biological conditions when all its members are included without investigating the degree of tolerance of each genus and species. Thus, “percent of total abundance without Chironomidae” was selected for measuring the productivity and instream habitat quality, and this metric showed a high level of discriminatory power and significant relationships, especially with watershed and physical factors.

Commonly used community indices were considered as composition measures, among which “Shannon’s diversity index” was found to be the most suitable. This index generally decreases with increasing degradation of habitat quality and at a very low level, represents a stressed community that tends to be unstable. The value of this index ranges from 0 to 5, and is maximal when all species are evenly distributed in the most desirable environments [26]. However, its utility for nationwide bioassessment calls for a careful and standardized application because factors other than disturbance can affect this index, depending on the sampling design used (e.g., sampling method, sample size, and level of identification) [81]. Also, “Shannon diversity index” was often reported to be redundant because of its high correlation with the “number of taxa” [13]. Some difficulties also were raised in the application of Shannon’s diversity index due to its wide variation in unpolluted or intermediately disturbed sites and non-linear pattern of display with increasing pollution [82,83]. This study revealed that “Shannon’s diversity index” was satisfactory for our metric selection process despite of a strong relationship between them (*r *= 0.660, *p* < 0.01).

Trophic measures provide information on productivity, available food sources and trophic status in stream ecosystems, using relative abundance of functional feeding groups [26,84]. Because each functional feeding group is expected to occur in proportionately higher abundance during accumulation of particular food sources or in particular habitat types [51], trophic metrics show good responsiveness to environmental change. Two of the 11 candidate trophic metrics were incorporated as components of the multimetric index. Among these, “percent gatherer individuals” is a good indicator of organic pollution [13,65], but had significant correlations with physical variables rather than chemical ones. A greater abundance of collector–gatherers was generally found in streams containing a large amount of organic matters because of their preference for fine particulate organic matter on the streambed. Many pollution-tolerant species (e.g., Oligochaeta, Baetidae, some Ephemerellidae, and Chironomidae) were classified as gatherers and had been assigned in other bio-assessment methods as having tolerances ranging from moderate to high values [37,85,86]. The other trophic metric in our index was “the ratio of filterers and scrapers”. Both functional groups generally occupy the same habitat, which involves clinging onto coarse-sized particles to feed on suspended fine organic matter and periphyton, respectively. Therefore, substrate stability and heterogeneity could be estimated based on this metric [57]. Filterers and scrapers also tend to be more affected than gatherers by changes in riparian vegetation, sedimentation and suspended solids [87].

Initially, two metrics applied at the national scale [44,62] were considered for inclusion as tolerance measures in this study. The “ESB” metric has been used for assessment of overall environmental quality, and is calculated by combining the assigned ecological scores of the individual species present (from a score of 1 for tolerant species to a score of 4 for intolerant species), regardless of their abundance [40]. Thus, this metric is expected to decrease with increasing anthropogenic disturbance, and to be positively correlated with most richness measures. In contrast, the “KSI” metric tends to increase with increasing organic pollution. The KSI metric evaluated the reference sites as having good to excellent water quality, whereas there were large variation in this metric for the impaired sites, which ranged from good to poor condition. This was attributed to imprecise classification of the indicator groups, suggesting that further detailed research is necessary on the tolerance levels of individual indicators in relation to water quality status [37]. Nevertheless, the KSI metric showed good sensitivity in discriminating the reference and impaired sites, and was included as a core component in the multimetric index.

### 4.4. Comparisons with Other Multimetric Indices

Many studies have been recently conducted to develop various types of multimetric indices to evaluate their own ecosystem health and integrity. A majority of multimetric indices were based on small sample size [88,89,90], one stream/river watershed [89,91], or a part of stream environments (e.g., headwater streams [55,88,90] and larger river [35,56,92]). A few studies were performed on a broad scale, especially across the states or the country [16,93,94]. Such indices can expose some limitations for classifying the reference conditions and selecting appropriate metrics, when applied to other streams or much broader watersheds. At first, the reference sites chosen from small spatial scales may not well correspond with those from larger ones. It may lead to a downgrading of the overall biological integrity for most of sampling sites, due to disparate conditions such as steam size, hydrology, land use patterns and physico-chemical environments. Also, each metric component needs to be tested and thus validated for its good performance [16]. In this respect, our developed multimetric index, KB-IBI, has the significance of examining a nation-wide applicability from extensive dataset casting a variety of stream sizes and disturbance types.

In the index development procedure, metric selection is the most critical element to best demonstrate the biological responses to environmental degradation [13,15,30,94]. Abundant metrics have been suggested by the aid of the species diversity and abundance of benthic macroinvertebrates. Many multimetric indices previously developed mostly include eight to 12 metrics representing species richness, composition, tolerance, and trophic measures [88]. There were most commonly used metrics (e.g., number of taxa, percent EPT individuals, and Shannon’s diversity index) as proven high discriminatory power and good responsiveness to disturbance [56,68,88,90,91,95] in our study. However, other most metric components seem to be region-specific or partly suitable for the conditions of particular aquatic environments. For example, the metrics of Plecoptera richness and percent of shredders were more applicable to headwater streams [55,88,89,90,95], and Coleoptera richness, percent Diptera, and percent predators to larger rivers [35,56]. Such metrics based on particular taxa were unsatisfactory for KB-IBI by causing the low sensitivity and high variability because each taxon displayed different ecological characteristics and environmental relationships along with stream channels (Table 3). In addition, although trophic measures were reported to be partly or unreliable in some studies [16,96], trophic and tolerance-related metrics were suitable and effective for evaluating the condition of the Korean streams.

Taxonomic resolution is an important issue in biological assessments [16,88,94,97]. Many rapid bioassessment methods prefer family-level identification for rapidity, convenience, economical advantages [4,15,94]. However, it is often claimed because even if various species and genera belong to the same family group, they often exhibit different tolerance levels and ecological traits [37,76,77,78,79]. An identification to the lowest possible taxon provides the precise ecological data, thereby enabling to detect multiple stressors and discriminate more accurately the differences in biological integrity [6,16,68,88,97]. This was supported by that KB-IBI showed much higher correlation coefficients with physico-chemical variables than did Macroinvertebrate Integrity Score in Stream (MISS) previously proposed by using the same database as this study [94]. Therefore, a careful consideration for determining identification level should be given depending on the objective of bioassessments.

### 4.5. Application of the KB-IBI for Stream Health Assessment

The suitability and robustness of a newly developed bio-assessment method requires to be confirmed with the field validation. Thus, the next phase of this study will be to test the applicability of the KB-IBI in the different streams. In a previous study we used a similar IBI-type multimetric index based on benthic macroinvertebrate communities; the index comprised eight metrics including five having the same properties as those included among the KB-IBI metrics. Although we do not provide the test results here, this index showed apparent sensitivity and strong discriminatory power in assessing the biological integrity of streams [98]. This IBI-type multimetric index clearly differentiated overall biotic integrity (with low levels of variance) at various sites from the headwaters to downstream in a stream affected by increased sedimentation and nutrient enrichment. In the same study the KSI, which was developed with indicator species in relation to BOD concentration [37], did not clearly identify the effect of physical alteration of the stream habitat on macroinvertebrate communities, and also showed substantial variation among sites. The newly developed KB-IBI, in combination with assessment of benthic macroinvertebrates, may enable differentiation of physical and chemical disturbances, and may be useful in future studies measuring the long-term status of streams and the effectiveness of various remediation methods. It may also provide a useful reference for bio-assessment methods developed in other countries. Consequently, our next study will investigate bio-assessment using the KB-IBI.

## 5. Conclusions

Deterioration of freshwater resources has long been a major concern of human society when the aim is to preserve and maintain balanced biological communities and sustainable stream ecosystem health. The ecological significance of aquatic ecosystems and societal concerns for the sustainable use of water resources in Korea and other countries require the development of more powerful tools to assess the biological integrity and health of stream ecosystems. Development of a suitable index for this purpose is critical for improving river management. We have developed a multimetric index based on benthic macroinvertebrate communities, using a nation-wide database covering 720 study sites in Korean streams. Eight metrics selected from an initial total of 34 candidates were incorporated into the final index (the KB-IBI; Korean Benthic macroinvertebrate Index of Biological Integrity). The index was able to distinguish reference and impaired sites, and showed significant correlations with all environmental variables considered in the study. The KB-IBI provides a tool for assessment of the biotic integrity of Korean streams, and is comparable to other multimetric indices developed in other geographic regions and a reasonable tool applicable to future studies assessing the long-term status of streams and the effectiveness of various remediation methods. Future research will refine the constituting metrics of the index, and thereby to improve its sensitivity and robustness. 
